# Oncogenic Network and Hub Genes for Natural Killer/T-Cell Lymphoma Utilizing WGCNA

**DOI:** 10.3389/fonc.2020.00223

**Published:** 2020-03-05

**Authors:** Huijiao Liu, Mei Liu, Hua You, Xiru Li, Xiangdong Li

**Affiliations:** ^1^Beijing Advanced Innovation Center for Food Nutrition and Human Health, College of Biological Sciences, China Agricultural University, Beijing, China; ^2^Affiliated Cancer Hospital & Institute of Guangzhou Medical University, Guangzhou, China; ^3^Department of Pathology, General Hospital of PLA, Beijing, China; ^4^Department of General Surgery, The 301th Hospital of PLA, Beijing, China; ^5^State Key Laboratory of Agrobiotechnology, College of Biological Sciences, China Agricultural University, Beijing, China

**Keywords:** natural killer/T-cell lymphoma (NKTCL), weight gene co-expression network analysis (WGCNA), co-expression network, LMO3, GRB14

## Abstract

Natural killer (NK)/T-cell lymphoma (NKTCL) is a subtype of non-Hodgkin lymphoma with aggressive progression and poor prognosis. The molecular mechanisms of NKTCL have not been well-studied. Herein, we revealed the lymphoma-associated dysregulated genes and signaling pathways or biological processes in NKTCL. We characterized that the extracellular matrix (ECM) receptor interaction pathway and T-cell receptor signaling pathway were the main dysregulated pathways in NKTCL by Gene Ontology (GO) analysis and pathway enrichment analysis. By using weighted gene co-expression network analysis (WGCNA), the gene co-expression network of NKTCL (SRP049695) was constructed, and hub genes (*LMO3, GRB14*) were identified. In addition, another Gene Expression Omnibus (GEO) dataset (GSE69406) was used to validate these hub genes. Furthermore, these hub genes were identified and validated by survival analysis (GSE90597). These results provided novel insights into the pathogenesis of NKTCL. Of particular interest, LMO3 and GRB14 might be potential oncoproteins and biomarkers for the diagnosis and treatment of NKTCL.

## Introduction

Natural killer (NK)/T-cell lymphoma (NKTCL) is a rare but aggressive lymphoid malignancy with poor prognosis and could be subdivided into two subtypes: nasal type and extra-nasal type (1). Still, NKTCL does not have a standard classification and treatment. Five years of overall survival (OS) of nasal type and extra-nasal type are 42 and 9%, respectively (2). So far, the pathogenesis of NKTCL remains unclear.

Recent works have achieved some progress in NKTCL by using array-based technologies. Somatic alterations, including TP53, DDX3X, and STAT3 mutations, have been identified in NKTCL (3, 4). TP53 functions as a tumor suppressor gene, which mainly leads cell cycle to arrest at the G1 phase. TP53 mutations are found in 20–60% of NKTCL (5, 6). DDX3X is an ATP-dependent RNA helicase and plays an important role in the nucleus by regulating transcription. A larger-scale research revealed a high frequent alteration (20%) of DDX3X in NKTCL (3). STAT3 and STAT5B were found mutated in about 12% frequency (7). Some single genes, such as surviving, *Aurora Kinase A (AURKA), C-MYC, EZH2, RUNX3*, were found to be deregulated in NKTCL (8–12). Several studies have provided evidence that the Janus kinase (JAK)/signal transducer and activator of transcription (STAT), platelet-derived growth factor (PDGF), NOTCH-1, nuclear factor (NF)-κB pathways in NKTCL were dysregulated (9, 13, 14). Genes involved in core co-expression network in NKTCL still need to be studied.

In previous work, most researchers focused on the screening of the most differential genes. Weighted gene co-expression network analysis (WGCNA) is widely used to explore the huge and complex relationships among genes across microarray or RNA sequence data (15). WGCNA provides a convenient and effective solution for screening core genes that could be potential biomarkers for clinical prognosis and treatment (16, 17).

In our study, WGCNA was first used to analyze the hub genes of NKTCL samples mined from the SRA database. Two other GEO databases were used to validate the results. Our findings may provide a new idea of the basic research of NKTCL and beneficial to the clinical diagnosis and treatment.

## Materials and Methods

### Data Collection and Preprocessing

Three normal NK normal control and 15 NKTCL case (Supplementary Table 1) RNA-Seq raw data files were downloaded from the National Center for Biotechnology Information (NCBI) Sequence Read Archive (SRA) with the ID SRP049695. TrimGalore (http://www.bioinformatics.babraham.ac.uk/projects/trim_galore/) method was used to filter low-quality raw reads as follows: (1) removal of primers sequencing; (2) removal of sequences of low quality in terminal; (3) removal of sequences <35 bp. Clean reads were aligned with HISAT2 to the USCS hg19 human genome assembly. mRNA and lncRNA quantitative expressions [fragments per kilobase million (FPKM)] were performed by Cufflinks (http://cole-trapnell-lab.github.io/cufflinks).

### Differentially Expressed Genes Screening

The Cuffdiff was used to screen the differentially expressed genes (DEGs) between normal NK cells and NKTCL cases. The DEGs threshold was set as follows: (1) *p* < 0.05; (2) log_2_ (fold change) > 1 or log_2_ (fold change) <-1; (3) *q* < 0.05. ggplot2 package in R was used to show the heat map and volcano maps.

### Gene Ontology Annotations and Kyoto Encyclopedia of Genes and Genomes Pathway Analyses

Gene Ontology (GO) analysis was performed to show the unique biological significance based on differentially expressed genes. The Kyoto Encyclopedia of Genes and Genomes (KEGG) database was carried out to find out the important pathway. The ClusterProfiler packages (18) in R was applied to analysis and demonstrated GO annotations and KEGG pathway.

### Gene Set Enrichment Analysis

Expression dataset from SRP049695 was converted to the tab-delimited GCT format as follows: The first column of the GCT file contains gene symbols. The second column was filled with “NA.” Subsequent columns were filled with each sample's expression value. The following operations were carried out according to the protocol (http://www.gsea-msigdb.org/gsea/) (19, 20).

### Protein–Protein Interaction Network Building

Differentially expressed mRNAs (fold change >2 or fold change < 0.5, *P* < 0.05) were taken into the Search Tool for the Retrieval of Interacting Genes/Proteins (STRING). The confidence score was set at 0.9. The Molecular Complex Detection (MCODE) was used to analyze the core modules of the protein–protein interaction (PPI) network.

### Co-expression Network Construction

WGCNA package of R software was applied to uncover the correlation among genes. Firstly, expression data of DEGs was input into R software to inspect good genes and samples, SRR1648324 and SRR1648323 were excluded from the analysis due to the poor quality. The power of β was set at 14 to ensure a scale-free network. The minimum number of module genes was set at 30. The hierarchical clustering dendrogram summarized the Gene modules with different colors. Heat map and topological overlap matrix (TOM) plot were used to visualize the module structure. The threshold of output to Cytoscape was set at 0.6.

### Hub Gene Selection and Validation

Gene network files exported from WGCNA analysis were input into Cytoscape software. The MCODE plugin of Cytoscape was used to calculate K-core value of each gene. GSE69406 dataset was used to validate the expression of the hub genes. GSE90597 dataset was used to plot the Kaplan–Meier survival curve in ggplot2 of R software.

## Results

### Overview of the Transcriptomes of Natural Killer/T-Cell Lymphoma

**Figure 1:**
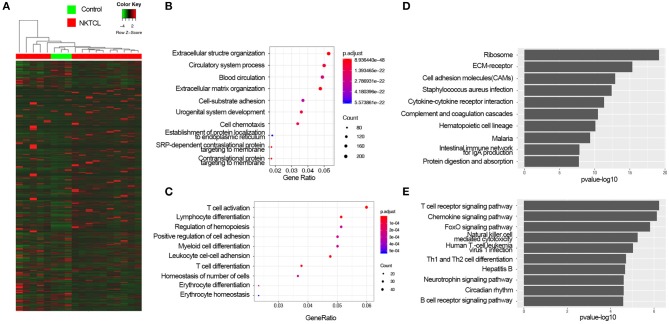
The expression profiles of natural killer T-cell lymphoma (NKTCL). **(A)** Heat map of all mRNAs detected by RNA-Seq. **(B)** Top 10 Gene Ontology (GO) terms of upregulated differentially expressed genes (DEGs) with fold change >2 and *P* <0.05 top 10 enrichment gene ratio of biological processes. **(C)** The Kyoto Encyclopedia of Genes and Genomes (KEGG) analysis of upregulated DEGs with top 10 enrichment P-value. **(D)** Top 10 GO terms of downregulated DEGs with fold change >2 and *P* <0.05. **(E)** KEGG analysis of downregulated DEGs with top 10 enrichment P-value.

In order to elucidate the molecular pathogenesis of NKTCL, heat maps of all mRNAs were shown in Figure 1A. To assess differential gene expressions between NKTCL and the normal NK cells, all genes were plotted in volcano plots. In total, 6,680 mRNAs displayed the differential expressions in NKTCL, including 5,664 upregulated and 1,016 downregulated mRNAs (Supplementary Figure 1A). Of note, 6,005 lncRNAs showed differential expressions, 4,968 lncRNAs were upregulated, and 1,037 lncRNAs were downregulated (Supplementary Figure 1B). Supplementary Figures 1C,D showed the dysregulated mRNAs or lncRNAs that were log_2_Foldchange | >10 in NKTCL.

### Gene Ontology and Kyoto Encyclopedia of Genes and Genomes Analysis of the Differentially Expressed Genes

To get an insight into the function of DEGs of NKTCL, the upregulated and downregulated DEGs were analyzed by enrichGO or enrichKEGG function of clusterProfiler packages in R software, respectively. GO analysis results were enriched in the biological process (BP), the upregulated DEGs significantly enriched in the extracellular structure organization, circulatory system process, blood circulation, extracellular matrix (ECM) organization (Figure 1B), and the downregulated DEGs significantly enriched in T-cell activation, lymphocyte differentiation, regulation of hemopoiesis, positive regulation of cell adhesion, myeloid cell differentiation, and leukocyte cell–cell adhesion (Figure 1C). KEGG analysis showed that the upregulated DEGs significantly were enriched in ribosome, ECM–receptor interaction, and cell adhesion molecules (CAMs) pathways (Figure 1D), and the downregulated DEGs were enriched in T-cell receptor signaling pathway, chemokine signaling pathway, and FoxO signaling pathway (Figure 1E). To illustrate the interactions of the upregulated and downregulated pathways in the progression of NKTCL, the top 10 of upregulated and downregulated enriched pathways and their genes were used to construct a network. The T-cell receptor signaling pathway and CAMs pathway were considered to be at the central network, ECM–receptor interaction pathway was found to be the linker between the upregulated pathways and the downregulated pathways (Supplementary Figure 2A). In order to further understand the changes of the pathogenesis in NKTCL, all DEGs, including the upregulated and downregulated genes, were put into KEGG pathway analysis, the molecular network constructed by top 20 of enriched pathways demonstrated that the phosphoinositide 3-kinase (PI3K)/Akt pathway is the most important signal in NKTCL (Supplementary Figure 2B).

### Gene Set Enrichment Analysis of Natural Killer/T-Cell Lymphoma

**Figure 2:**
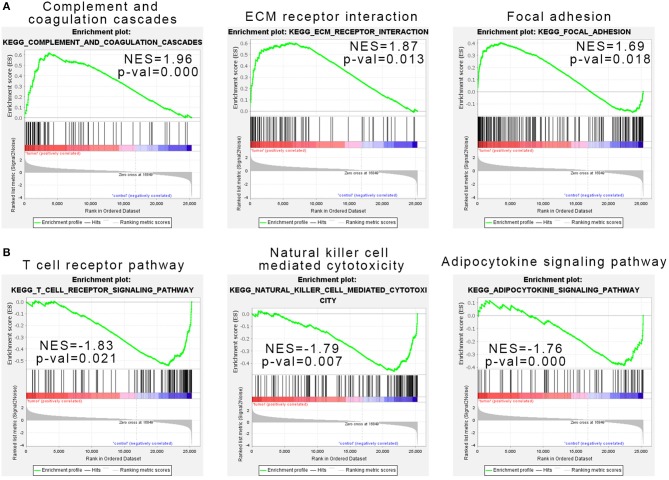
Gene set enrichment analysis indicated the Kyoto Encyclopedia of Genes and Genomes (KEGG) pathways in natural killer T-cell lymphoma (NKTCL). **(A)** NKTCL samples were correlated positively with gene signatures related to complement and coagulation cascades, extracellular matrix (ECM) receptor interaction, and focal adhesion pathways. **(B)** NKTCL samples were correlated negatively with gene signatures related to T-cell receptor pathway, natural killer cell-mediated cytotoxicity, and adipocytokine signaling pathway.

Considering that the number of genes input in GO and KEGG analysis is our artificially defined standard (*p* < 0.05 and |log_2_ (FC)|>1), the results may be different under different standards (such as *p* < 0.01 and |log_2_(FC)|>1). Here we used all expression data sets of SRP049695 for Gene Set Enrichment Analysis (GSEA) analysis, and the results show that complement and coagulation cascades, ECM receptor interaction, and focal adhesion pathways were enriched in the tumor samples (Figure 2A). T-cell receptor pathway, NK cell-mediated cytotoxicity, and adipocytokine signaling pathway were enriched in the control samples (Figure 2B).

### Protein–Protein Interaction of Natural Killer T-Cell Lymphoma

**Figure 3:**
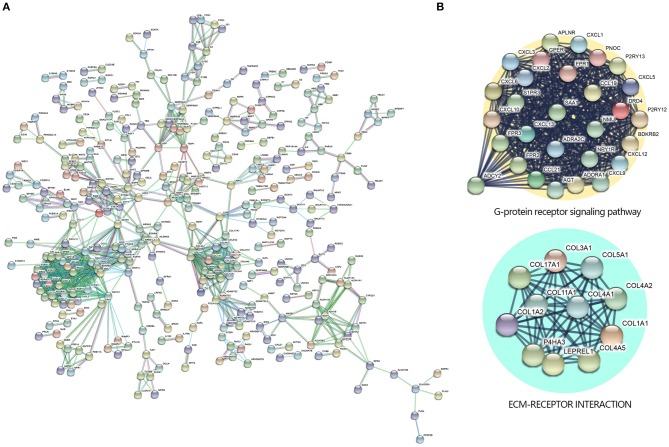
Protein–protein interaction (PPI) networks by Search Tool for the Retrieval of Interacting Genes/Proteins (STRING) Search Tool. **(A)** The PPI network was constructed by STRING based on differentially expressed genes (DEGs) with fold change >2 and *P* < 0.05 that were input into STRING, the confidence score was set at 0.9. **(B)** The 40 highest k-core genes make up two important subnetworks.

To further investigate the DEGs and the potential protein levels, the STRING database was applied for revealing the core PPI network. As shown in Figure 3A, 982 nodes and 1,076 edges constructed the core PPI network. The network data were reanalyzed by MCODE to get the k-core which reflected the importance of each gene. The 40 highest k-core genes make up two important networks, one is the G protein-coupled receptor signaling pathway, the other is ECM receptor signaling pathway (Figure 3B), which may suggest the underlying mechanism in the NKTCL pathogenesis.

### Weighted Gene Correlation Network Analysis of Natural Killer T-Cell Lymphoma

**Figure 4:**
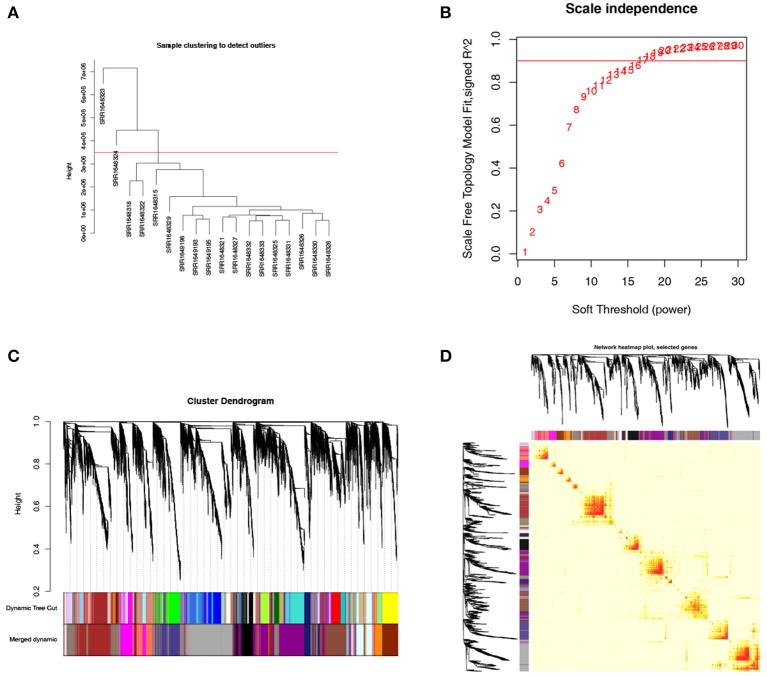
Weighted gene co-expression network analysis (WGCNA) of genes in natural killer T-cell lymphoma (NKTCL). **(A)** Cluster dendrogram displays the relationship between NKTCL samples. **(B)** Analysis of the scale-free fit index for various soft thresholding powers (β). **(C)** Hierarchical cluster tree showing co-expression modules identified by WGCNA. **(D)** Heat map plot show the topological overlap matrix (TOM) among all genes. Light color shows low overlap, and red color indicates higher overlap. The left side and the top side show the gene dendrogram and module assignment.

To clarify the key modules and genes in NKTCL, WGCNA was used to uncover the highly correlated genes and the co-expression networks of NKTCL. SRR1648323 and SRR1638324 samples were excluded from analysis after quality assessment (Figure 4A). The power of β was automatically set at 14 to ensure a scale-free network (Figure 4B). Gene modules were calculated, and the gray module represents genes that cannot be clustered into any other modules (Figure 4C). Finally, 18 gene modules were identified by the hierarchical clustering dendrogram. The interactions between gene modules were subsequently analyzed, and the TOM plot of a gene network was generated with the corresponding hierarchical clustering dendrogram and the modules (Figure 4D).

### Identifying the Hub Genes of Natural Killer T-Cell Lymphoma

**Figure 5:**
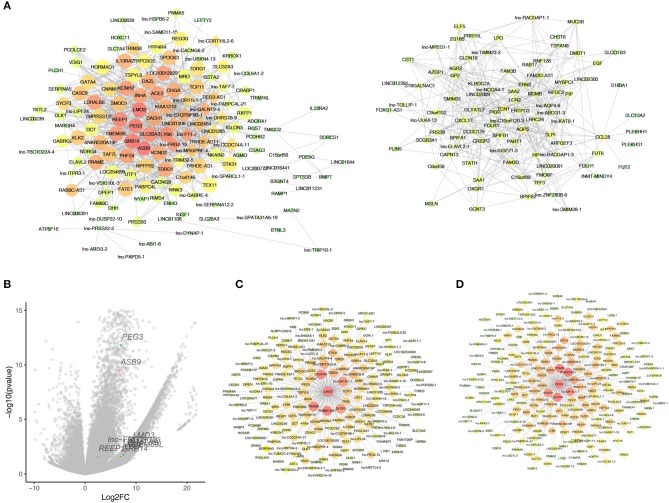
Identified hub genes in natural killer T-cell lymphoma (NKTCL). **(A)** Co-expression networks constructed by weighted gene co-expression network analysis (WGCNA). Circle represents mRNA; rhombus represents long non-coding RNA; line represents correlative relationship. Large sizes and red colors represent the high degrees and k-core scoring. **(B)** Top 10 score genes were plot in volcano with labels and different colors. **(C)** Genes that co-expressed with LMO3. **(D)** genes that co-expressed with PEG3.

As WGCNA generated a huge gene network, we narrowed the network by set edges weight >0.6 to localize the hub genes. As a result, 325 nodes and 6,900 edges were screened out for further analysis. Figure 5A showed two crucial subnetworks. The most important genes were constructed by the edges degree and k-core. The subnetwork contains mRNAs, such as *LMO3, PEG3, GRB14, ASB9*, and lncRNAs, such as lnc-FRG2-13, lnc-F11-2, lnc-GALNT9-4, LINC01206, probably constituting the core of the networks. To visualize these hub genes, a total of 57 genes (k-core >60) and the top 10 core genes were listed in the volcano plots (Figure 5B). We observed the highest fold changes in *LMO3* among these genes, and *PEG3* is one of the most significantly changed genes. Further, the co-expression genes of *LMO3* and *PEG3* were plotted in Figures 5C,D.

### Validation of Dysregulated Genes in Natural Killer T-Cell Lymphoma vs. the Normal Controls

**Figure 6:**
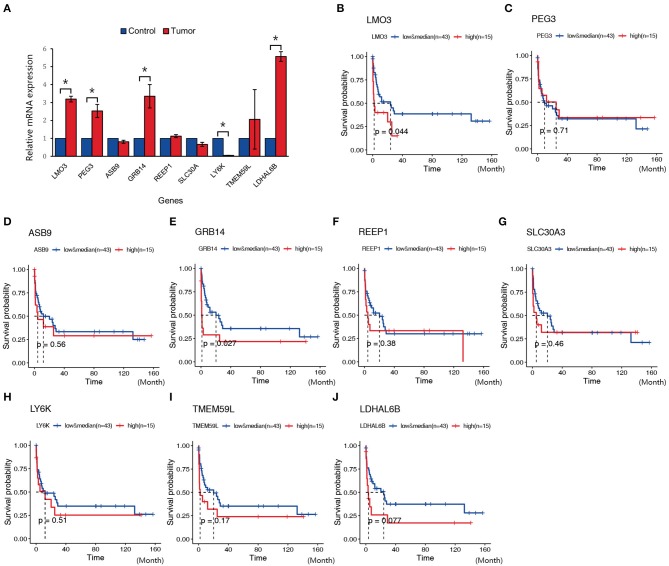
Expression and survival plot of genes in samples from other Gene Expression Omnibus (GEO) datasets or patients with natural killer T-cell lymphoma (NKTCL). **(A)** Expression of LMO3, PEG3, ASB9, GRB14, REEP1, SLC30A, LY6K, TMEM59L, and LDHAL6B in GEO datasets (GSE69406). **(B–J)** Kaplan–Meier survival curve of genes in GEO datasets (GSE90597); the red line represents the high expression group (top quarter of the median, *n* = 15), the blue line represents the median and low expression group (*n* = 43), the x-axis unit is month. Results are presented as the mean±SD (*P < 0.01).

In order to validate the possible key genes which screened from WGCNA, we explored the top 10 genes, namely, *LMO3, PEG3, ASB9, GRB14, REEP1, SLC30A3, LY6K, TMEM59L*, and *LDHAL6B*, in GEO database with the ID GSE69406, which contains three control cell line samples and five NKTCL samples. As a result, *LMO3, PEG3, GRB14*, and *LDHAL6B* were significantly upregulated in NKTCL, whereas *ASB9* and others displayed no significant changes between NKTCL and the controls (Figure 6A). To reveal the relationship between the expression of these genes and the survival rate, another GEO dataset with the ID GSE90597 was used to plot the Kaplan–Meier survival curve. Figures 6B–J showed the OS between the high expression group and the median/low expression group. As a result, the overexpression of LMO3 (*p* = 0.044) and GRB14 (*p* = 0.027) are significantly related to the shorter period of OS.

## Discussion

NKTCL is biologically heterogeneous and has a variable clinical course, which most commonly affects nasal cavity and sometimes involves extra-nasal organs such as the skin, gastrointestinal tract, and testis. Since NKTCL has distinct and massive necrotic lesions, it is hard to get enough specimens to clarify the molecular mechanism of NKTCL (21). To understand the molecular mechanism in NKTCL, we utilized three datasets from the SRA database and the GEO database. We identified that *LMO3* and *GRB14* were the hub genes of NKTCL and found that high expressions of *LMO3* and *GRB14* were related to the poor prognosis. Thus, LMO3 and GRB14 might be the potential biomarkers for the diagnosis and treatment of NKTCL.

Gene expression profiling studies had provided a method to explore the molecular mechanism of NKTCL. The *Homo sapiens* transcriptome or gene expression dataset (SRP049695) which we used in this study was first performed by Kucuk et al. (7) to find the mutations in NKTCL. Kucuk et al. (7) found that STAT3 and STAT5B were each mutated at 5.9% frequency (*n* = 51). Baytak et al. (22) focused on the dysregulated oncogenic lncRNA (MIR155HG) in NKTCL by analyzing the SRP049695 dataset. In this study, we found that ECM receptor interaction pathway was significant in KEGG (Figure 1D), PPI network (Figure 3), and GSEA (Figure 2A) analysis.

Conventional approaches for finding the core genes require a selection of hundreds of different genes, whereas WGCNA is particularly useful in summarizing many intra-modular hub genes that can be speculated for the diagnosis and prognosis of tumors (15, 23). In our study, *LMO3* and *GRB14* were screened as the hub genes of NKTCL by WGCNA. *LMO3* encoded a 17KD protein that belongs to a cysteine-rich LIM domain protein family. The protein family also contains LMO1 and LMO2. *LMO1* and *LMO2* are oncogenes that are recurrently translocated and overexpressed in T-cell acute lymphoblastic leukemia (T-ALL) (24). LMO3 could promote gastric cancer cell invasion and proliferation through Akt-mTOR and Akt-GSK3β signaling pathways (25). In hepatocellular carcinoma, LMO3 directly interacts with LATS1 and suppressing Hippo signaling to promote invasion and metastasis (26). LMO3 interacts with the DNA binding domain of P53 to inhibit its activity (27). To validate the relation of LMO3 to NKTCL, another GEO dataset (GSEGSE69406) was applied in this study. It was shown that *LMO3* was overexpressed in tumor compared to the controls (Figure 5A). Moreover, the high expression of *LMO3* was related to the low survival rate in GSE90597 dataset (Figure 5B). As a result, it is reasonable to speculate that *LMO3* could be a potential oncogene and can promote the progress of NKTCL. To further validate the hypothesis of the oncogenic potency and the molecular action of *LMO3* in NKTCL, further *in vitro, in vivo*, and clinical studies are needed and will be carried out in our future study.

GRB14 interacts with insulin receptors and shows its inhibitory effect function on receptor tyrosine kinase signaling (28). Overexpression of GRB14 facilitated STAT3 and Akt phosphorylation (29). Considering that continuous JAK–STAT pathway activation is a major feature of NKTCL, it would be very interesting to study whether GRB14 promotes the progress of NKTCL by activating STAT3 (3, 7). Besides, NKTCL patients have amino acid metabolism disorders, such as alanine, aspartate, and glutamate (30). Asparaginase is the most widely used antimetabolite agent in NKTCL (31). To clarify whether GRB14 is involved in the NKTCL oncogenesis by affecting metabolism has also become very meaningful. Experiments on the relationship between GRB14 and NKTCL metabolism are being conducted in our ongoing study. However, the shortcoming of this study is that it is not able to verify the hub genes in clinical samples.

In summary, we used the bioinformatics method to identify and validate hub genes of NKTCL; LMO3 and GRB14 may be the potential targets for diagnosis and treatment of NKTCL.

## Data Availability Statement

Publicly available datasets were analyzed in this study. This data can be found here: https://www.ncbi.nlm.nih.gov/sra/?term=SRP049695; https://www.ncbi.nlm.nih.gov/geo/query/acc.cgi?acc=GSE69406; https://www.ncbi.nlm.nih.gov/geo/query/acc.cgi?acc=GSE90597.

## Author Contributions

HL, HY, and XiaL conceived and designed the study and contributed to writing of the manuscript. HL, XirL, and XiaL performed the analysis procedures. HL and ML analyzed the results. HY and ML contributed analysis tools. All authors reviewed the manuscript.

### Conflict of Interest

The authors declare that the research was conducted in the absence of any commercial or financial relationships that could be construed as a potential conflict of interest.
