# Supplementary material for: Oncogenic Network and Hub Genes for Natural Killer/T-Cell Lymphoma Utilizing WGCNA
Source: Front Oncol. 2020 Mar 5;10:223. doi: 10.3389/fonc.2020.00223 (PMC7066115; doi:10.3389/fonc.2020.00223)
Supplement: Supplementary file 1 [file Data_Sheet_1.pdf]

Supplementary Material

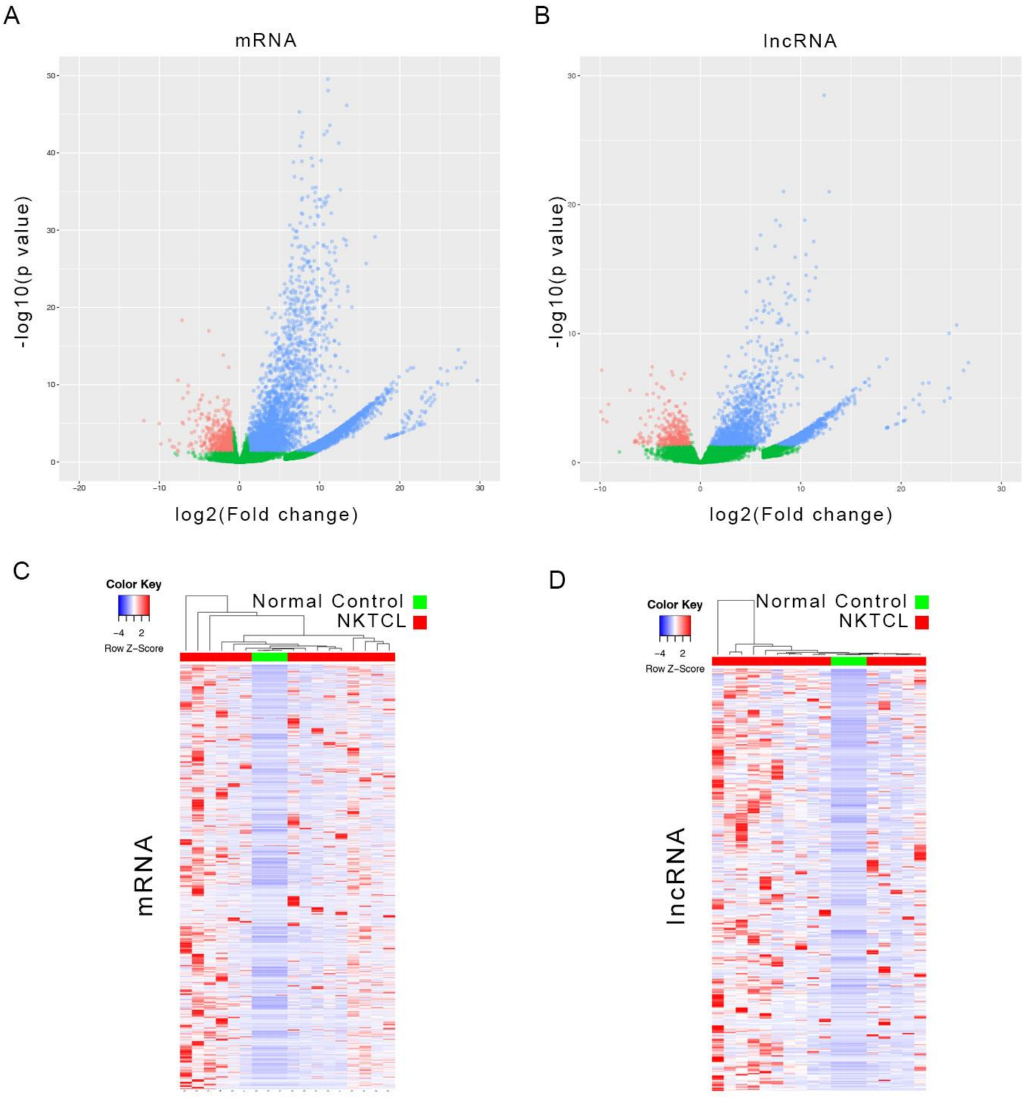

Supplementary Figure 1. (A). Volcano of all mRNAs in NKTCL, red and blue plots show the significantly changed mRNAs with change ≥2.0 and P <0.05. **(B).** Volcano of all long non-coding RNAs in NKTCL, red and blue plots show the significantly changed mRNAs with change ≥2.0 and P <0.05. **(C).** heatmap of mRNAs with log2 fold change ≥10. **(D).** heatmap of long non-coding RNAs with log2 fold change ≥10.

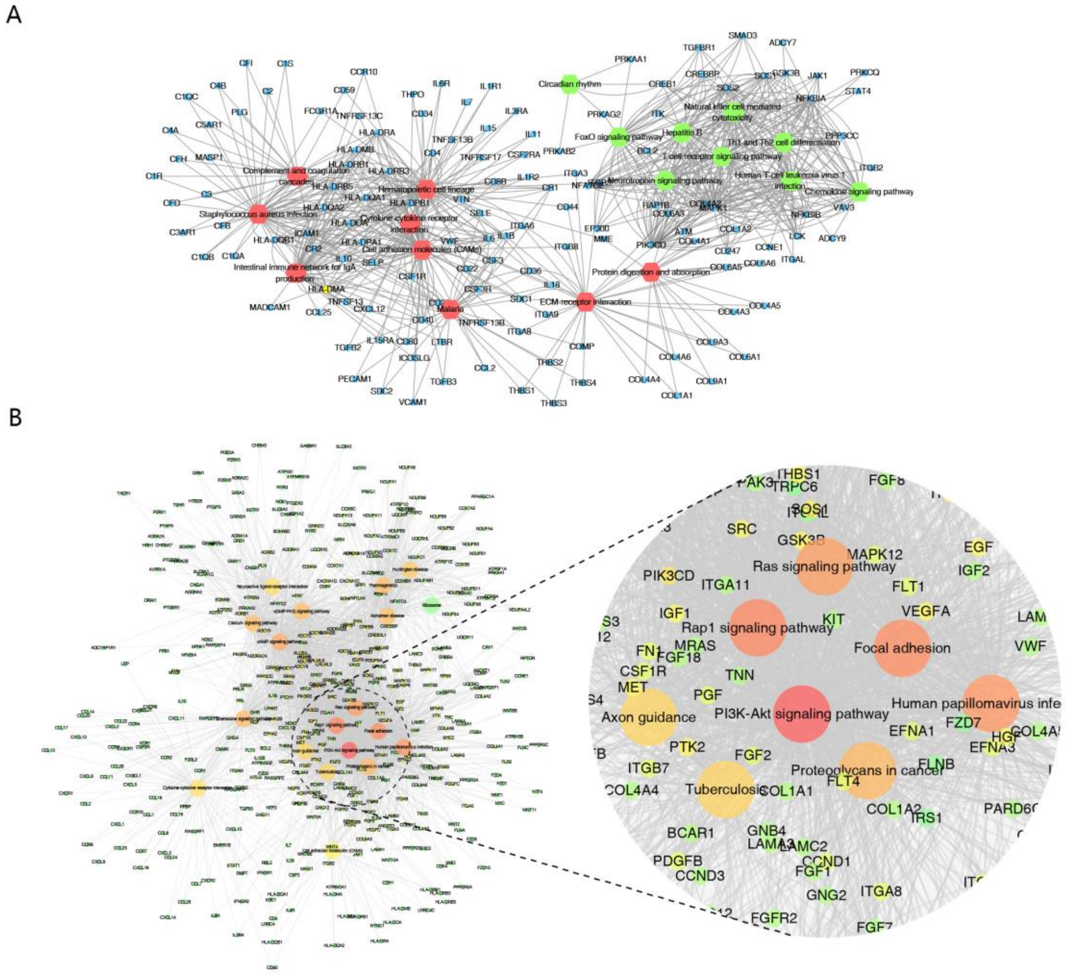

Supplementary Figure 2. (A). Interaction among up or down regulated KEGG pathways. The hexagons filled with red color were up regulated KEGG pathway and with green color were down regulated KEGG pathway; ellipses represent the link mRNAs between two pathways. **(B).** Interaction and overlapping of associated molecules among top 20 dysregulated significant pathways. The node size and color represent the center of the network. The higher the node k-core, the deeper the color and the larger the node.

Supplementary Table 1

Characteristics of NKTCL cases and normal control cells used in this study.

Type	SRR ID	Biopsy site	Sex	EBV	TCR arragement
NKTCL	1648315	Lymph node	M	+	TCRαβ +
NKTCL	1648318	Nasal	M	+	germline
NKTCL	1648321	Nasal	M	+	Germline
NKTCL	1648322	Nasal	F	+	Germline
NKTCL	1648323	Lymph node	M	+	TCRαβ +
NKTCL	1648324	Lymph node	M	NA	Germline
NKTCL	1648325	Lymph node	M	+	Germline
NKTCL	1648326	Small bowel	F	+	Germline
NKTCL	1648327	Nasal	F	+	Germline
NKTCL	1648328	Testis	M	+	Germline
NKTCL	1648329	NA	F	NA	germline
NKTCL	1648330	NA	M	NA	germline
NKTCL	1648331	Testis	M	+	germline
NKTCL	1648332	Nasal	F	+	germline
NKTCL	1648333	nasal	M	+	germline
Normal NK Cells	1648193	NKCOD12			
Normal NK Cells	1648195	PBNK48h			
Normal NK Cells	1648196	Resting NK			
